# Sex-Specific Differences in the Relationship between Insulin Resistance and Adiposity Indexes in Children and Adolescents with Obesity

**DOI:** 10.3390/children8060449

**Published:** 2021-05-26

**Authors:** Valeria Calcaterra, Elvira Verduci, Laura Schneider, Hellas Cena, Annalisa De Silvestri, Sara Vizzuso, Federica Vinci, Chiara Mameli, Gianvincenzo Zuccotti

**Affiliations:** 1 Pediatrics and Adolescentology Unit, Department of Internal Medicine, University of Pavia, 27100 Pavia, Italy; fede90vinci@gmail.com; 2 Department of Pediatrics, “Vittore Buzzi” Children’s Hospital, 20154 Milan, Italy; elviraverduci@unimi.it (E.V.); lauraschneider@asst-fbf-sacco.it (L.S.); saravizzuso@asst-fbf-sacco.it (S.V.); chiaramameli@unimi.it (C.M.); gianvincenzozuccotti@unimi.it (G.Z.); 3 Department of Health Sciences, University of Milan, 20146 Milan, Italy; 4 Laboratory of Dietetics and Clinical Nutrition, Department of Public Health, Experimental and Forensic Medicine, University of Pavia, 27100 Pavia, Italy; hellas.cena@unipv.it; 5 Clinical Nutrition and Dietetics Service, Unit of Internal Medicine and Endocrinology, ICS Maugeri IRCCS, 27100 Pavia, Italy; 6 Biometry & Clinical Epidemiology, Scientific Direction, Fondazione IRCCS Policlinico San Matteo, 27100 Pavia, Italy; a.desilvestri@smatteo.pv.it; 7 Pediatric Unit, Fondazione IRCCS Policlinico San Matteo, 27100 Pavia, Italy; 8 Department of Biomedical and Clinical Science “L. Sacco”, University of Milan, 20157 Milan, Italy

**Keywords:** sex-specific differences, insulin resistance, adiposity, children, adolescents, obesity

## Abstract

New indexes of adiposity have been introduced to evaluate body-fat distribution and cardiometabolic risk. However, data on the correlation between Insulin Resistance (IR) and these new indexes are limited. We therefore evaluated the relationship between IR and adiposity indexes in children and adolescents with obesity, focusing on gender differences. We retrospectively enrolled 586 patients with obesity (10.80 ± 2.63; 306F/279M). As adiposity indexes we considered body mass index (BMI), BMI-z score, WC, waist-to-height ratio (WHtR), a body shape index (ABSI), triponderal mass index (TMI), visceral adiposity index (VAI) and conicity index (ConI). The homeostasis model assessment for insulin resistance (HOMA-IR), HOMA of percentage β-cell function (HOMA-β), quantitative insulin sensitivity check index (QUICKI), and triglyceride and glucose index (TyG-index) were measured and recorded as IR surrogates. In both sexes, WC and VAI significantly correlated with all IR measurements (*p* < 0.001). BMI significantly correlated (*p* < 0.001) with all IR parameters except for the TyG-index in females. Fat mass and TMI correlated with IR parameters only in females, BMI-z score with IR markers except for HOMA-β in males, WHtR with HOMA-β in both sexes (*p* < 0.05), free fat mass with HOMA-IR and QUICKI only in females (*p* < 0.01), ConI correlated with the TyG index in females (*p* = 0.01). Tryglicerides and SBP were correlated with all IR measurements (*p* < 0.001), in both sexes. Correlations between different sex parameters were significantly more evident in middle puberty. The relationship between IR surrogates and obesity indexes is influenced by gender in pediatrics. Sex-specific differences in obesity-related complications should be considered in preventive intervention decision-making.

## 1. Introduction

Insulin resistance (IR) is a crucial factor contributing to the pathogenetic mechanism of several disorders, including type 2 diabetes mellitus (DMT2), hypertension, dyslipidemia, cardiovascular disease, and metabolic syndrome [1,2,3]. IR represents the earliest manifestation of dysmetabolism in children [4,5].

Euglycemic-hyperinsulinemic clamp is the gold standard method for determining insulin sensitivity. However, it is invasive, time intensive and not practical in the pediatric age [6,7]. Consequently, several indirect methods have been proposed for clinical studies and epidemiological evaluations. In particular, surrogate measures, based on fasting levels of insulin and glucose are validated tools that simplify IR measurement and are used in epidemiological studies and in clinical practice [8,9,10]; these include the homeostasis model assessment for insulin resistance (HOMA-IR), HOMA of percentage β-cell function (HOMA-β) and quantitative insulin sensitivity check index (QUICKI). The triglyceride and glucose index (TyG-index) may also be useful for IR prediction in large-scale studies or screening of populations at high risk of diabetes, as they are more sensitive in recognizing IR compared with HOMA-IR [11,12].

Body-fat distribution is also a critical player in IR development [13,14,15]. Body mass index (BMI), waist circumference (WC) and or waist-to-height ratio (WHtR) are usually considered good markers of the dysmetabolic profile [16,17,18,19,20,21,22]. Recently, new indexes of obesity, including body shape index (ABSI), triponderal mass index (TMI), conicity index (ConI) and the visceral adiposity index (VAI) have been introduced to evaluate body-fat distribution and cardiometabolic risk [23,24,25,26,27,28,29,30,31,32].

Limited data on the correlation between IR and these new indexes of adiposity have been reported in the pediatric age [28,32]. Therefore, we analyzed the association of IR surrogates and adiposity indexes in children and adolescents with obesity. Considering that IR is partly attributed to sex differences in fat mass, we focused the evaluation on sex differences. The relationship between IR and other cardiometabolic risk factors was also considered.

## 2. Materials and Methods

### 2.1. Patients

We retrospectively enrolled 586 Caucasian children and adolescents (376 females and 279 males) with obesity (BMI-z score ≥ 2 according to the World Health Organization), aged 10.80 ± 2.63 years (range 6–18 years) referred to the Outpatient Clinics of the Vittore Buzzi Children’s Hospital, Milano, Fondazione IRCCS Policlinico S. Matteo, Pavia, and the San Paolo University Hospital of Milan. Patients were referred by their general practitioner or primary care pediatrician between January 2015 and December 2020. Known secondary obesity conditions, use of any medications, and concomitant chronic or acute illnesses were considered exclusion criteria.

### 2.2. Physical Examination and Adiposity Indexes

In all participants, height, weight, pubertal stage, WC, BMI, adiposity indexes, and body composition were measured. Total and HDL-cholesterol levels as well as diastolic (DBP) and systolic blood pressure (SBP) measurements were also collected.

Height, weight, pubertal stage, WC measurement, DBP and SBP were performed as reported elsewhere [12]. As previously reported, pubertal stages were classified as follows, Prepubertal stage = Tanner Stage 1; Middle puberty = Tanner Stages 2–3; Late puberty = Tanner Stages 4–5 [12].

BMI was calculated as body weight (kilograms) divided by height (meters squared) and was transformed into BMI-z scores using World Health Organization (WHO) reference values [33].

In addition to BMI, BMI-z score, and WC, adiposity indexes, including WHtR, ABSI, TMI, VAI, ConI were considered and calculated as follows:

- WHtR = WC/Ht
- ABSI = 1000 × WC × Wt ^−2/3^ × Ht^5/6^ [34]
- TMI = weight (kg)/height (m)^3^ [29]
- ConI = WC/(0.109 × (Wt/Ht)^0.5^) [35]
- VAI [36]

Male = [WC/(39.68 + (1.88 × BMI))] × (TG/1.03) × (1.31/HDL-C);

Female = [WC/(36.58 + (1.89 × BMI))] × (TG/0.81) × (1.52/HDL-C)

### 2.3. Biochemical Evaluation and Insulin Resistance Measurements

At enrollment, in addition to the collection of historical data and a physical examination, a biochemical evaluation was performed. Patients underwent a blood draw in a fasting state between 8:30 a.m. and 9:00 a.m. and plasma glucose, insulin, triglycerides (TG), total, and HDL-cholesterol were analyzed the same morning by standard methods.

As IR surrogates we considered the following:

- HOMA-IR calculated as insulin resistance = (insulin × glucose)/22.5 [8];
- TyG-index calculated as ln[fasting triglycerides (mg/dL) × fasting plasma glucose (mg/dL)/2]) [37];
- HOMA of percentage β-cell function (HOMA-β) calculated as HOMA-β% = (FIRI × 20)/(FPG − 3.5) [8];
- Quantitative Insulin sensitivity Check Index (QUICK index) calculated as the inverse of the sum of the logarithms of fasting insulin and fasting glucose: 1/(log(fasting insulin μU/mL) + log(fasting glucose mg/dL)) [38].

### 2.4. Statistical Analysis

Quantitative variables were described as the mean and standard deviation when normally distributed (Shapiro–Wilks’s test). Comparisons based on sex were made with the Student T test for independent samples. Relationships between the indexes, were assessed with the Pearson correlation coefficient. A *p*-value < 0.05 was considered statistically significant.

## 3. Results

Anthropometric features, adiposity indexes and indirect IR markers in the patients according to sex, are summarized in Table 1.

### 3.1. Adiposity Indexes

Adiposity indexes according to sex and pubertal stages are reported in Table 1. The BMI-z score did not differ in males and females; in both sexes it was higher in prepuberty than in middle and late puberty (*p* < 0.001).

Compared to females, males had higher WC (*p* < 0.01), ABSI (*p* < 0.01), ConI (*p* < 0.01) and lower VAI and TMI (*p* < 0.01) values, Table 1.

The difference in WC was influenced by puberty in both sexes, with higher values in puberty compared to the prepubertal period (*p* < 0.001). ABSI in prepubertal females was higher compared to girls in middle and late puberty (*p* = 0.001). In males, no differences related to pubertal stage were noted (*p* = 0.27). In both males and females, TMI and ConI did not differ between puberty groups (*p* > 0.05), Table 1.

VAI in pubertal males was higher compared to boys in middle and late puberty (*p* = 0.001); no significant differences related to pubertal stage were noted in females (*p* = 0.04), Table 1.

Fat mass and free fat mass did not differ in females and males (*p* > 0.05), Table 1. Fat mass was influenced by puberty in both sexes, with higher values in pubertal stages compared to prepubertal stages (*p* < 0.01). Free fat mass in prepubertal females was higher compared to girls in middle and late puberty (*p* = 0.001), while no differences were related to pubertal stage in boys (*p* = 0.54), Table 1.

### 3.2. Indirect IR Markers

Concerning IR indexes, lower HOMA-β (*p* < 0.001) levels were detected in males compared to females. No other differences in IR indexes were noted between sex groups (*p* > 0.05), Table 1. The differences in all IR parameters were influenced by puberty in both sexes, with higher values in pubertal patients compared to patients in the prepubertal period (*p* < 0.001).

### 3.3. Correlation between IR Measurements and Adiposity Indexes

As reported in Table 2, in both sexes, WC and VAI significantly correlated with all indirect IR measurements (*p* < 0.001).

Other correlations between adiposity indexes and IR parameters varied according to sex. In particular, BMI was correlated (*p* < 0.001) with all indirect IR parameters except for the TyG-index in females. Fat mass and TMI correlated with all IR parameters only in females; BMI z-score correlated with IR markers except for HOMA-β in males (*p* > 0.05); WC/H was not correlated with IR indexes except with HOMA-β in both sexes (males *p* = 0.01; females *p* = 0.04); Free fat mass was related to HOMA-IR and QUICKI only in females (*p* < 0.01); ConI was only correlated with the TyG-index in females (*p* = 0.01). ABSI was not correlated with IR measurements.

As reported in Table 2, all significant correlations were influenced by puberty in both males and females. For BMI, BMI-z score, WC, Fat mass, free fat mass, VAI, TMI, and ConI, the correlation with IR parameters lost significance in late puberty, particularly in females, Table 2.

The differences between females and males were more evident in middle puberty, in particular for fat mass, free fat mass, VAI, TMI and Con I (*p* < 0.01).

### 3.4. Correlation between IR Measurements and Cardiometabolic Parameters

As shown in Table 3, in both sexes, triglycerides and SBP significantly correlated with all indirect IR measurements (*p* < 0.001).

Total cholesterol levels did not correlate with IR indexes, except for the TyG-index in both sexes (*p* < 0.001). DBP was not correlated with the TyG-index in females or males. HDL-cholesterol correlated with all indirect IR (*p* < 0.001) parameters, except for HOMA-IR and QUICKI in females.

Correlations between parameters and the differences between the sexes were significantly more evident in middle puberty, Table 3.

## 4. Discussion

We reported sex-specific differences in the relationship between IR and adiposity indexes, such as BMI, BMI-z score, WC, fat mass, free fat, TMI, ConI, ABSI, VAI, in children and adolescents with obesity. Sex differences were also noted in the correlation between IR markers and associated cardiometabolic risk factors, including lipidic profile and blood pressure. Puberty, body-fat distribution, and body composition may play a role in the differences between the sexes.

Childhood obesity is a multisystem condition associated with several metabolic diseases [39]. IR is an early manifestation of dysmetabolism in pediatric patients, and is also considered a precursor and risk factor for other cardiovascular diseases [1,2,3,4,5]. IR is characterized by an inappropriate compensatory insulin secretory response, which leads to hyperinsulinemia [40].

In this study, we observed sex-specific differences in the relationship between IR and adiposity indexes in children and adolescents with obesity. Gender differences were also noted in the correlation between IR markers and associated cardiometabolic risk factors. Puberty, body-fat distribution, and body composition may play a role in these differences.

Some simple methods, including HOMA-IR, HOMA-β, QUICKI, have been validated for use in epidemiological and clinical studies to detect IR and β-cell function and predict the development of diabetes in a non-diabetic population [8]. Recently, the TyG-index has also been proposed for IR estimation [11,12].

Several factors are known to contribute to IR development in patients with obesity. Body composition, including distribution of body fat and accumulation of excess fat in certain depots is a crucial player in the development of IR to the extent that visceral fat accumulation has been shown to be a determinant of IR and cardiometabolic risk [16,17,18,19,20,21,22,23,24,25,26,27,28,29,30,31,32,41]. In pediatrics, pubertal changes in insulin sensitivity have been reported to contribute to IR [42].

BMI, WC, and WHtR have been widely used as indexes of obesity, because of their practical application. However, new parameters such as ABSI, VAI, TMI and the C-Index have been proposed to better evaluate body-fat distribution [16,17,18,19,20,21,22,23,24,25,26,27,28,29,30,31,32,41]. In our population significant differences were noted between these parameters according to sex and pubertal stage, supporting the concept of differences in adipose tissue distribution according to gender and hormonal influence [43].

Data on the correlations between new markers of adiposity and IR in pediatrics are limited [28,32]. We describe here for the first time, sex-specific differences with respect to these correlations. In both males and females, we observed that WC and VAI were closely associated with IR indexes, confirming that these parameters are valuable indicators of adipose tissue dysfunction [43] and are better predictors of impaired glucose metabolism compared to BMI and other anthropometric measurements [44,45]. In support of this concept, there is evidence that visceral fat is associated with adipokine production which play a key role in insulin sensitivity [44]. Additionally, the capacity of IR indexes to estimate muscle or central insulin sensitivity should be considered [8,9,10,11,12].

Other indexes highlighted different correlations with IR parameters according to sex, even though IR indexes were similar in males and females. In particular, some measurements, including total fat mass and TMI, were correlated with IR parameters only in females. These results are supported by the well-known sex differences in adipose tissue distribution [43]. Due to differences in the timing of pubertal development between females and males, the role of sexual hormones was also considered [42]. Moreover, different adipose tissue functions according to gender, such as adipokine secretion related to insulin sensitivity, cannot be excluded [43]. For example, it has been reported that developmental genes may contribute to depot- and sex-specific properties of adipose tissue [43].

In this study, ABSI was not correlated with IR indexes, showing that this parameter is a poor predictor of IR [46]. Bouchi et al. have reported that ABSI related to abdominal vs. peripheral fat ratio is positively correlated with visceral adipose depot and is supposed to be BMI-independent [47]. ABSI has also been shown to be significantly associated with cardiometabolic risk markers in the pediatric population with overweight or obesity [28]; as reported this risk may be influenced by other variables, such as hypertension [41].

As with ABSI, ConI was a poor predictor of IR considering parameters based on fasting levels of insulin and glucose. On the contrary, in females it was related to the TyG-index, supporting a role in dysmetabolism linked to hypertension and dyslipidemia rather than to IR [48].

In this study, sex-specific differences were also observed in the correlation between IR surrogates and associated cardiometabolic risk factors, such as total cholesterol, HDL-cholesterol, and DBP. These data support the idea that additional genetic and lifestyle factors including dietary patterns and exercise, may also influence IR and the dysmetabolic profile [1,5,49].

Western diets poor in fruit and vegetables and rich in animal-based sources of proteins affect insulin secretion and reduce IR by insufficient intake of antioxidant food compounds; negative microbiota interactions and increased metabolic acid load that have also been linked to IR and impaired glucose homeostasis [50].

Data on the impact of sex-specific differences on health are scarce in the pediatric age. The study of sex-specific differences in obesity-related complications, such as prevalence, severity of progression, and symptom presentation represent crucial information for prevention strategies in order to improve public health. Additionally, the critical importance of sex differences as modulators of drug response have been reported [51]. Thus, these differences should be considered for tailored treatments also in pediatrics.

Study limitations The study has some limitations that should be considered. First, even though the gold standard for IR determination is the euglycaemic-hyperinsulinaemic clamp technique, we used the indirect measurement of IR since the clamp is difficult to use in routine clinical practice or large epidemiologic studies, particularly in children. Secondly, we only evaluated correlations between IR and adiposity indexes; however, the role of physical activity and diet in insulin sensitivity, should be considered. Additionally, in females with adipomastia normal breast development with female Tanner classification may be difficult to evaluate. However, the Tanner scale is the gold standard in rating pubertal development in clinical practice.

Finally, sex hormone evaluations were not included in the analysis and their influence on IR cannot be excluded.

## 5. Conclusions

The relationship between IR surrogates and obesity indexes is influenced by gender in pediatrics and puberty represents the period in which these correlations were more evident. Sex-specific differences in obesity-related complications are meaningful in the detection process of pre-disease stage and in therapeutic intervention planning for children and adolescents.

## Figures and Tables

**Table 1:** Indexes of obesity and insulin resistance and metabolic parameters of the patients according to sex and pubertal stages.

Parameters	All	Females (*n* = 306)	Males (*n* = 279)	*p* *
Age (years)	10.80 ± 2.63	11.4 ± 3.0	11.2 ± 2.90	0.98
Pubertal stages				
Tanner 1	145	89	56	
Tanner 2–3	375	174	201	<0.001
Tanner 4–5	65	43	22	
Body mass index (kg/m^2^)	28.36 ± 4.40	28.46 ± 4.84	28.26 ± 3.87	0.58
Tanner 1	25.13 ± 3.18 **	24.88 ± 2.87 **	25.54 ± 3.62 **	
Tanner 2–3	28.95 ± 4.15	29.28 ± 4.72	28.67 ± 3.60	
Tanner 4–5	32.18 ± 3.52	32.53 ± 3.80	31.48 ± 2.84	
Body mass index z score	2.73 ± 0.73	2.72 ± 0.72	2.74 ± 0.74	0.78
Tanner 1	3.07 ± 1.04 **	3.03 ± 1.04 **	3.12 ± 1.04 **	
Tanner 2–3	2.65 ± 0.57	2.63 ± 0.50	2.67 ± 0.62	
Tanner 4–5	2.42 ± 0.42	2.44 ± 0.45	2.39 ± 0.37	
Waist circumference (cm)	87.77 ± 11.28	86.49 ± 11.22	89.14 ± 11.18	0.005
Tanner 1	77.62 ± 7.68 **	77.56 ± 7.72 **	77.72 ± 7.67 **	
Tanner 2–3	90.10 ± 9.74	88.76 ± 9.84	91.13 ± 9.55	
Tanner 4–5	97.73 ± 10.32	96.50 ± 9.96	100.03 ± 10.82	
Waist circumference/height ratio	0.58 ± 0.11	0.58 ± 0.10	0.57 ± 0.12	0.38
Tanner 1	0.59 ± 0.10	0.60 ± 0.09 **	0.58 ± 0.10	
Tanner 2–3	0.57 ± 0.13	0.56 ± 0.15	0.58 ± 0.12	
Tanner 4–5	0.60 ± 0.06	0.60 ± 0.06	0.59 ± 0.06	
Fat mass (%)	37.28 ± 5.85	37.42 ± 5.69	36.78 ± 6.0	0.06
Tanner 1	35.28 ± 4.88 **	35.86 ± 4.59 **	34.35 ± 5.22 **	
Tanner 2–3	37.69 ± 5.77	37.87 ± 5.78	37.57 ± 5.78	
Tanner 4–5	39.15 ± 7.10	41.06 ± 5.86	35.52 ± 7.96	
Free Fat mass (%)	60.98 ± 9.8	60.25 ± 9.5	61.77 ± 10.2	0.09
Tanner 1	61.85 ± 12.26	62.48 ± 9.46 **	60.90 ± 15.67	
Tanner 2–3	61.12 ± 8.79	59.65 ± 9.78	62.24 ± 7.65	
Tanner 4–5	58.81 ± 9.76	5818 ± 8.12	60.04 ± 12.53	
Visceral Adiposity Index	3.31 ± 2.93	3.87 ± 3.00	2.71 ± 2.71	<0.001
Tanner 1	2.82 ± 2.16	3.52 ± 2.42	1.73 ± 0.95 **	
Tanner 2–3	3.46 ± 3.26	4.18 ± 3.38	2.84 ± 3.03	
Tanner 4–5	3.60 ± 2.25	3.34 ± 2.22	4.04 ± 2.28	
Body shape index	0.078 ± 0.006	0.077 ± 0.006	0.079 ± 0.006	0.005
Tanner 1	0.079 ± 0.007 **	0.080 ± 0.005 **	0.078 ± 0.008	
Tanner 2–3	0.078 ± 0.005	0.076 ± 0.005	0.079 ± 0.005	
Tanner 4–5	0.075 ± 0.006	0.075 ± 0.006	0.077 ± 0.005	
Triponderal mass index	19.32 ± 2.60	19.61 ± 2.93	19.01 ± 2.13	0.004
Tanner 1	19.45 ± 2.47	19.42 ± 2.47	19.50 ± 2.50	
Tanner 2–3	19.20 ± 2.66	19.56 ± 3.18	18.90 ± 2.07	
Tanner 4–5	19.67 ± 2.47	20.18 ± 2.75	18.68 ± 1.39	
Conicity index	1.25 ± 0.09	1.24 ± 0.09	1.26 ± 0.09	0.003
Tanner 1	1.25 ± 0.10	1.25 ± 0.08	1.23 ± 0.12 **	
Tanner 2–3	1.25 ± 0.08	1.23 ± 0.08	1.27 ± 0.08	
Tanner 4–5	1.23 ± 0.10	1.22 ± 0.10	1.25 ± 0.09	
Homeostasis model assessment for insulin resistance	3.42 ± 2.42	3.58 ± 2.52	3.24 ± 2.29	0.08
Tanner 1	2.36 ± 1.56 **	2.62 ± 1.73 **	1.96 ± 1.14 **	
Tanner 2–3	3.72 ± 2.38	4.02 ± 2.63	3.45 ± 2.11	
Tanner 4–5	4.12 ± 3.40	3.84 ± 2.96	4.64 ± 4.12	
Triglyceride and Glucose index	8.10 ± 0.46	8.10 ± 0.46	8.09 ± 0.53	0.68
Tanner 1	7.97 ± 0.48 **	8.05 ± 0.47 **	7.85 ± 0.49 **	
Tanner 2–3	8.15 ± 0.49	8.17 ± 0.45	8.12 ± 0.53	
Tanner 4–5	8.09 ± 0.52	7.94 ± 0.45	8.35 ± 0.54	
HOMA of percentage β-cell function	193.09 ± 76.67	205.71 ± 78.49	179.11 ± 72.21	<0.001
Tanner 1	156.10 ± 63.77 **	168.6 ± 69.57 **	136.90 ± 48.22 **	
Tanner 2–3	203.53 ± 76.48	222.84 ± 79.40	186.13 ± 69.48	
Tanner 4–5	216.36 ± 77.74	211.45 ± 65.91	225.3 ± 96.73	
Quantitative insulin sensitivity check index	3.05 ± 0.28	3.08 ± 0.27	3.03 ± 0.28	0.05
Tanner 1	2.91 ± 0.27 **	2.95 ± 0.28 **	2.83 ± 0.24 **	
Tanner 2–3	3.11 ± 0.26	3.14 ± 0.26	3.08 0.26	
Tanner 4–5	3.13 ± 0.28	3.14 ± 0.27	3.17 ± 0.30	
Fasting blood glucose (mg/dL)	81.74 ± 8.92	80.68 ± 9.34	82.89 ± 8.29	0.002
Tanner 1	80.75 ± 8.21	80.58 ± 7.65	81.01 ± 9.07	
Tanner 2–3	82.20 ± 8.79	80.70 ± 9.68	83.47 ± 7.71	
Tanner 4–5	81.21 ± 10.88	80.51 ± 11.09	82.59 ± 10.59	
Insulin (mU/mL)	16.75 ± 10.46	17.71 ± 10.93	15.67 ± 9.81	0.01
Tanner 1	11.71 ± 7.44 **	13.09 ± 8.35 **	9.56 ± 5.09 **	
Tanner 2–3	18.17 ± 10.28	19.79 ± 11.31	16.72 ± 9.04	
Tanner 4–5	19.90 ± 13.41	18.80 ± 11.44	21.91 ± 16.51	
Total cholesterol (mg/dL)	155.31 ± 27.61	152.94 ± 25.92	157.89 ± 29.18	0.03
Tanner 1	154.65 ± 26.06	154.52 ± 27.61	154.85 ± 23.87 **	
Tanner 2–3	156.21 ± 27.77	153.65 ± 24.01	158.47 ± 30.57	
Tanner 4–5	151.50 ± 30.04	146.80 ± 29.66	160.45 ± 29.35	
HDL-cholesterol (mg/dL)	47.10 ± 11.10	47.25 ± 11.45	46.91 ± 10.71	0.71
Tanner 1	48.89 ± 12.52 **	48.52 ± 12.79	49.46 ± 1217 **	
Tanner 2–3	47.04 ± 10.58	47.28 ± 11.30	46.83 ± 9.92	
Tanner 4–5	43.40 ± 9.80	44.57 ± 8.61	41.18 ± 11.63	
Tryglicerides (mg/dL)	92.29 ± 58.56	92.21 ± 49.38	92.37 ± 67.22	0.97
Tanner 1	80.64 ± 40.82 **	87.34 ± 44.76 **	70.33 ± 31.57 **	
Tanner 2–3	96.83 ± 65.06	97.90 ± 53.03	95.89 ± 74.17	
Tanner 4–5	91.38 ± 48.07	78.02 ± 38.32	116.95 ± 54.41	
Systolic Blood Pressure (mmHg)	111.96 ± 14.20	111.86 ± 12.49	112.07 ± 15.92	0.43
Tanner 1	105.41 ± 12.20 **	105.38 ± 12.35 **	105.47 ± 11.81 **	
Tanner 2–3	113.39 ± 14.28	114.29 ± 11.29	112.70 ± 16.39	
Tanner 4–5	117.95 ± 13.20	115.86 ± 12.36	122.04 ± 14.11	
Diastolic Blood Pressure (mmHg)	63.62 ± 9.49	63.68 ± 8.69	63.50 ± 10.33	0.82
Tanner 1	60.13 ± 7.58 **	60.18 ± 8.21 **	60.05 ± 6.47 **	
Tanner 2–3	63.97 ± 9.66	64.25 ± 8.22	63.67 ± 10.74	
Tanner 4–5	69.06 ± 9.41	68.55 ± 8.65	70.04 ± 10.88	

* *p* females vs. males; ** significant difference (*p* < 0.05) between Tanner stages.

**Table 2:** Correlation between insulin resistance measurements and adiposity indexes.

Adiposity Indexes	Indirect Insulin Resistance Measurements											
	HOMA-IR			TyG-Index			HOMA-β			QUICK Index		
	All	Females	Males	All	Females	Males	All	Females	Males	All	Females	Males
Body mass index	r = 0.41	r = 0.42	r = 0.39	r = 0.14	r = 0.11	r = 0.18	r = 0.45	r = 0.49	r = 0.41	r = 0.41	r = 0.43	r = 0.39
	*p* < 0.001	*p* < 0.001	*p* < 0.001	*p* < 0.001	*p* = 0.05	*p* = 0.001	*p* < 0.001	*p* < 0.001	*p* < 0.001	*p* < 0.001	*p* < 0.001	*p* < 0.001
Tanner 1	r = 0.40	r = 0.49	r = 0.26	r = 0.16	r = 0.23	r = 0.16	r = 0.36	r = 0.42	r = 0.41	r = 0.31	r = 0.42	r = 0.25
	*p* < 0.001	*p* < 0.001	*p* = 0.04	*p* = 0.05	*p* = 0.03	*p* = 0.24	*p* < 0.001	*p* < 0.001	*p* = 0.01	*p* < 0.001	*p* < 0.001	*p* = 0.05
Tanner 2–3	r = 0.40	r = 0.46	r = 0.30	r = 0.08	r = 0.13	r = 0.04	r = 0.45	r = 0.55	r = 0.30	r = 0.36	r = 0.43	r = 0.28
	*p* < 0.001	*p* < 0.001	*p* < 0.001	*p* = 0.08	*p* = 0.07	*p* = 0.56	*p* < 0.001	*p* < 0.001	*p* < 0.001	*p* < 0.001	*p* < 0.001	*p* < 0.001
Tanner 4–5	r = 0.13	r = −0.05	r = 0.57	r = 0.10	r = −0.02	r = 0.53	r = −0.01	r = −0.24	r = 0.43	r = 0.13	r = −0.13	r = 0.48
	*p* = 0.28	*p* = 0.72	*p* < 0.01	*p* = 0.43	*p* = 0.88	*p* = 0.01	*p* = 0.93	*p* = 0.11	*p* = 0.04	*p* = 0.27	*p* = 0.93	*p* = 0.02
Body mass index z score	r = 0.19	r = 0.24	r = 0.15	r = 0.18	r = 0.19	r = 0.18	r = 0.08	r = 0.19	r = −0.02	r = 0.21	r = 0.27	r = 0.15
	*p* < 0.001	*p* < 0.001	*p* = 0.01	*p* < 0.001	*p* < 0.001	*p* = 0.002	*p* = 0.03	*p* < 0.001	*p* = 0.68	*p* < 0.001	*p* < 0.001	*p* = 0.01
Tanner 1	r = 0.34	r = 0.43	r = 0.21	r = 0.24	r = 0.20	r = 0.33	r = 0.23	r = 0.39	r = −0.05	r = 0.35	r = 0.44	r = 0.24
	*p* < 0.001	*p* < 0.001	*p* = 0.11	*p* < 0.01	*p* = 0.06	*p* = 0.01	*p* < 0.01	*p* < 0.001	*p* = 0.71	*p* < 0.001	*p* < 0.001	*p* = 0.06
Tanner 2–3	r = 0.36	r = 0.47	r = 0.29	r = 0.25	r = 0.27	r = 0.25	r = 0.10	r = 0.36	r = 0.08	r = 0.36	r = 0.47	r = 0.31
	*p* < 0.001	*p* < 0.001	*p* < 0.001	*p* < 0.001	*p* < 0.001	*p* < 0.001	*p* < 0.01	*p* < 0.001	*p* = 0.21	*p* < 0.001	*p* < 0.001	*p* < 0.001
Tanner 4–5	r = 0.18	r = −0.08	r = 0.66	r = 0.12	r = 0.002	r = 0.42	r = 0.003	r = −0.29	r = 0.47	r = 0.16	r = −0.02	r = 0.58
	*p* = 0.14	*p* = 0.61	*p* < 0.001	*p* = 0.32	*p* = 0.98	*p* = 0.04	*p* = 0.97	*p* = 0.06	*p* = 0.02	*p* = 0.19	*p* = 0.89	*p* < 0.01
Waist circumference	r = 0.41	r = 0.43	r = 0.42	r = 0.20	r = 0.15	r = 0.25	r = 0.46	r = 0.53	r = 0.45	r = 0.43	r = 0.44	r = 0.44
	*p* < 0.001	*p* < 0.001	*p* < 0.001	*p* < 0.001	*p* = 0.008	*p* < 0.001	*p* < 0.001	*p* < 0.001	*p* < 0.001	*p* < 0.001	*p* < 0.001	*p* < 0.001
Tanner 1	r = 0.35	r = 0.50	r = 0.04	r = 0.09	r = 0.22	r = −0.07	r = 0.31	r = 0.41	r = 0.13	r = 0.30	r = 0.41	r = 0.11
	*p* < 0.001	*p* < 0.001	*p* = 0.75	*p* = 0.29	*p* = 0.03	*p* = 0.60	*p* < 0.001	*p* < 0.001	*p* = 0.33	*p* < 0.001	*p* < 0.001	*p* = 0.39
Tanner 2–3	r = 0.39	r = 0.48	r = 0.34	r = 0.14	r = 0.16	r = 0.14	r = 0.44	r = 0.60	r = 0.38	r = 0.39	r = 0.47	r = 0.34
	*p* < 0.001	*p* < 0.001	*p* < 0.001	*p* < 0.01	*p* = 0.03	*p* = 0.04	*p* < 0.001	*p* < 0.001	*p* < 0.001	*p* < 0.001	*p* < 0.001	*p* < 0.001
Tanner 4–5	r = 0.16	r = −0.08	r = 0.41	r = 0.42	r = 0.25	r = 0.54	r = 0.22	r = −0.06	r = 0.36	r = 0.13	r = −0.01	r = 0.29
	*p* = 0.20	*p* = 0.59	*p* < 0.001	*p* < 0.001	*p* = 0.12	*p* < 0.001	*p* = 0.08	*p* = 0.71	*p* = 0.09	*p* = 0.31	*p* = 0.94	*p* = 0.17
Waist circumference/height ratio	r = 0.08	r = 0.07	r = 0.10	r = 0.07	r = 0.07	r = 0.06	r = 0.07	r = 0.11	r = 0.14	r = 0.07	r = 0.06	r = 0.10
	*p* = 0.05	*p* = 0.18	*p* = 0.09	*p* = 0.08	*p* = 0.17	*p* = 0.26	*p* = 0.07	*p* = 0.04	*p* = 0.01	*p* = 0.07	*p* = 0.26	*p* = 0.08
Tanner 1	r = 0.14	r = 0.22	r = −0.05	r = 0.009	r = 0.04	r = −0.06	r = 0.16	r = 0.23	r = 0.01	r = 0.09	r = 0.13	r = −0.009
	*p* = 0.09	*p* = 0.03	*p* = 0.66	*p* = 0.90	*p* = 0.67	*p* = 0.63	*p* = 0.04	*p* = 0.03	*p* = 0.90	*p* = 0.28	*p* = 0.23	*p* = 0.944
Tanner 2–3	r = 0.08	r = 0.10	r = 0.09	r = 0.08	r = 0.11	r = 0.06	r = 0.12	r = 0.16	r = 0.15	r = 0.09	r = 0.10	r = 0.11
	*p* = 0.08	*p* = 0.15	*p* = 0.17	*p* = 0.11	*p* = 0.14	*p* = 0.34	*p* = 0.01	*p* = 0.02	*p* = 0.02	*p* = 0.07	*p* = 0.15	*p* = 0.11
Tanner 4–5	r = 0.312	r = −0.05	r = 0.40	r = 0.37	r = −0.31	r = 0.58	r = 0.11	r = −0.04	r = 0.34	r = 0.17	r = −0.08	r = 0.34
	*p* = 0.32	*p* = 0.74	*p* = 0.06	*p* = 0.003	*p* = 0.05	*p* < 0.01	*p* = 0.38	*p* = 0.78	*p* = 0.12	*p* = 0.17	*p* = 0.61	*p* = 0.11
Fat mass	r = 0.18	r = 0.27	r = 0.05	r = 0.12	r = 0.13	r = 0.10	r = 0.18	r = 0.25	r = 0.08	r = 0.19	r = 0.30	r = 0.04
	*p* < 0.001	*p* < 0.001	*p* = 0.38	*p* = 0.006	*p* = 0.02	*p* = 0.10	*p* < 0.001	*p* < 0.001	*p* = 0.18	*p* < 0.001	*p* < 0.001	*p* = 0.48
Tanner 1	r = 0.26	r = 0.31	r = 0.12	r = 0.23	r = 0.18	r = −0.25	r = 0.20	r = 0.23	r = 0.06	r = 0.23	r = 0.27	r = 0.11
	*p* < 0.01	*p* < 0.01	*p* = 0.40	*p* < 0.01	*p* = 0.10	*p* = 0.07	*p* = 0.02	*p* = 0.03	*p* = 0.67	*p* < 0.01	*p* = 0.01	*p* = 0.42
Tanner 2–3	r = 0.19	r = 0.34	r = 0.01	r = 0.05	r = 0.13	r = −0.003	r = 0.16	r = 0.28	r = 0.03	r = 0.14	r = 0.37	r = −0.06
	*p* < 0.001	*p* < 0.001	*p* = 0.88	*p* = 0.29	*p* = 0.08	*p* = 0.96	*p* < 0.01	*p* < 0.001	*p* = 0.66	*p* < 0.001	*p* < 0.001	*p* = 0.37
Tanner 4–5	r = −0.14	r = −0.20	r = −0.002	r = 0.07	r = −0.13	r = 0.34	r = −0.07	r = −0.18	r = 0.06	r = −0.04	r = −0.11	r = 0.10
	*p* = 0.30	*p* = 0.721	*p* = 0.99	*p* = 0.60	*p* = 0.43	*p* = 0.13	*p* = 0.60	*p* = 0.29	*p* = 0.80	*p* = 0.73	*p* = 0.452	*p* = 0.65
Free Fat mass	r = −0.18	r = −0.22	r = −0.004	r = −0.04	r = −0.002	r = −0.08	r = −0.03	r = −0.12	r = 0.08	r = −0.10	r = −0.19	r = 0.007
	*p* = 0.004	*p* < 0.001	*p* = 0.95	*p* = 0.30	*p* = 0.97	*p* = 0.22	*p* = 0.43	*p* = 0.06	*p* = 0.22	*p* = 0.03	*p* = 0.002	*p* = 0.91
Tanner 1	r = 0.01	r = −0.01	r = 0.03	r = −0.04	r = 0.13	r = −0.22	r = 0.05	r = 0.0001	r = 0.11	r = 0.08	r = 0.10	r = 0.05
	*p* = 0.86	*p* = 0.88	*p* = 0.79	*p* = 0.41	*p* = 0.27	*p* = 0.12	*p* = 0.56	*p* = 0.99	*p* = 0.43	*p* = 0.37	*p* = 0.41	*p* = 0.70
Tanner 2–3	r = −0.23	r = −0.31	r = −0.06	r = −0.04	r = −0.05	r = −0.01	r = −0.08	r = −0.15	r = 0.07	r = −0.19	r = −0.30	r = −0.03
	*p* < 0.001	*p* < 0.001	*p* = 0.44	*p* = 0.41	*p* = 0.51	*p* = 0.82	*p* = 0.13	*p* = 0.06	*p* = 0.38	*p* < 0.01	*p* < 0.001	*p* = 0.66
Tanner 4–5	r = 0.04	r = −0.02	r = −0.09	r = −0.03	r = −0.04	r = −0.07	r = 0.13	r = 0.12	r = 0.14	r = −0.05	r = −0.16	r = −0.02
	*p* = 0.72	*p* = 0.87	*p* = 0.70	*p* = 0.82	*p* = 0.78	*p* = 0.74	*p* = 0.32	*p* = 0.46	*p* = 0.56	*p* = 0.64	*p* = 0.35	*p* = 0.92
VAI	r = 0.26	r = 0.21	r = 0.32	r = 0.77	r = 0.79	r = 0.80	r = 0.32	r = 0.27	r = 0.32	r = 0.29	r = 0.24	r = 0.33
	*p* < 0.001	*p* < 0.001	*p* < 0.001	*p* < 0.001	*p* < 0.001	*p* < 0.001	*p* < 0.001	*p* < 0.001	*p* < 0.001	*p* < 0.001	*p* < 0.001	*p* < 0.001
Tanner 1	r = 0.30	r = 0.33	r = 0.38	r = 0.80	r = 0.87	r = 0.88	r = 0.39	r = 0.38	r = 0.12	r = 0.38	r = 0.33	r = 0.41
	*p* < 0.001	*p* = 0.001	*p* < 0.01	*p* < 0.001	*p* < 0.001	*p* < 0.001	*p* < 0.001	*p* < 0.001	*p* = 0.35	*p* < 0.001	*p* = 0.001	*p* = 0.001
Tanner 2–3	r = 0.27	r = 0.22	r = 0.31	r = 0.78	r = 0.77	r = 0.81	r = 0.31	r = 0.23	r = 0.32	r = 0.28	r = 0.23	r = 0.30
	*p* < 0.001	*p* < 0.01	*p* < 0.001	*p* < 0.001	*p* < 0.001	*p* < 0.001	*p* < 0.001	*p* < 0.01	*p* < 0.001	*p* < 0.001	*p* = 0.002	*p* < 0.001
Tanner 4–5	r = 0.01	r = −0.10	r = 0.12	r = 0.84	r = 0.84	r = 0.88	r = 0.09	r = 0.10	r = 0.06	r = 0.07	r = 0.001	r = 0.15
	*p* = 0.93	*p* = 0.54	*p* = 0.58	*p* < 0.001	*p* < 0.001	*p* < 0.001	*p* = 0.46	*p* = 0.54	*p* = 0.79	*p* < 0.001	*p* = 0.99	*p* = 0.050
ABSI	r = −0.04	r = −0.05	r = −0.02	r = 0.07	r = 0.10	r = 0.04	r = −0.02	r = −0.004	r = −0.009	r = −0.05	r = −0.07	r = −0.02
	*p* = 0.24	*p* = 0.32	*p* = 0.68	*p* = 0.07	*p* = 0.07	*p* = 0.42	*p* = 0.56	*p* = 0.93	*p* = 0.87	*p* = 0.16	*p* = 0.21	*p* = 0.64
Tanner 1	r = −0.03	r = 0.02	r = −0.21	r = −0.12	r = −0.07	r = −0.21	r = −0.04	r = 0.01	r = −0.23	r = −0.07	r = −0.06	r = −0.16
	*p* = 0.65	*p* = 0.79	*p* = 0.10	*p* = 0.15	*p* = 0.47	*p* = 0.11	*p* = 0.56	*p* = 0.90	*p* = 0.08	*p* = 0.38	*p* = 0.58	*p* = 0.23
Tanner 2–3	r = −0.01	r = 0.01	r = 0.001	r = 0.13	r = 0.15	r = 0.15	r = 0.01	r = 0.07	r = 0.05	r = −0.001	r = 0.03	r = 0.01
	*p* = 0.76	*p* = 0.84	*p* = 0.98	*p* = 0.01	*p* = 0.04	*p* = 0.03	*p* = 0.84	*p* = 0.31	*p* = 0.48	*p* = 0.99	*p* = 0.65	*p* = 0.84
Tanner 4–5	r = 0.04	r = −0.01	r = 0.10	r = 0.38	r = 0.34	r = 0.35	r = 0.21	r = 0.25	r = 0.13	r = 0.06	r = 0.04	r = 0.05
	*p* = 0.72	*p* = 0.92	*p* = 0.065	*p* < 0.01	*p* = 0.03	*p* = 0.10	*p* = 0.10	*p* = 0.12	*p* = 0.53	*p* = 0.62	*p* = 0.77	*p* = 0.82
TMI	r = 0.23	r = 0.30	r = 0.07	r = 0.07	r = 0.11	r = 0.02	r = 0.25	r = 0.33	r = 0.09	r = 0.18	r = 0.26	r = −0.03
	*p* < 0.001	*p* < 0.001	*p* = 0.06	*p* = 0.06	*p* = 0.04	*p* = 0.65	*p* < 0.001	*p* < 0.001	*p* = 0.10	*p* < 0.001	*p* < 0.001	*p* = 0.55
Tanner 1	r = 0.18	r = 0.29	r = −0.02	r = 0.03	r = 0.06	r = −0.003	r = 0.20	r = 0.25	r = 0.13	r = 0.12	r = 0.22	r = −0.04
	*p* = 0.02	*p* < 0.01	*p* = 0.83	*p* = 0.72	*p* = 0.54	*p* = 0.97	*p* = 0.01	*p* = 0.01	*p* = 0.31	*p* = 0.14	*p* = 0.03	*p* = 0.74
Tanner 2–3	r = 0.30	r = 0.38	r = 0.13	r = 0.11	r = 0.17	r = 0.04	r = 0.35	r = 0.47	r = 0.13	r = 0.23	r = 0.33	r = 0.09
	*p* < 0.001	*p* < 0.001	*p* = 0.06	*p* = 0.03	*p* = 0.02	*p* = 0.56	*p* < 0.001	*p* < 0.001	*p* = 0.06	*p* < 0.001	*p* < 0.001	*p* = 0.20
Tanner 4–5	r = 0.09	r = −0.03	r = 0.58	r = 0.01	r = 0.003	r = 0.58	r = −0.11	r = −0.31	r = 0.41	r = 0.15	r = 0.07	r = 0.58
	*p* = 0.48	*p* = 0.84	*p* < 0.01	*p* = 0.87	*p* = 0.98	*p* = 0.004	*p* = 0.38	*p* = 0.704	*p* = 0.05	*p* = 0.23	*p* = 0.63	*p* < 0.01
ConI	r = 0.08	r = 0.09	r = 0.09	r = 0.12	r = 0.15	r = 0.10	r = 0.17	r = 0.11	r = 0.09	r = 0.07	r = 0.07	r = −0.08
	*p* = 0.05	*p* = 0.11	*p* = 0.13	*p* = 0.003	*p* = 0.01	*p* = 0.07	*p* = 0.003	*p* = 0.06	*p* = 0.10	*p* = 0.08	*p* = 0.18	*p* = 0.15
Tanner 1	r = 0.04	r = 0.16	r = −0.20	r = −0.09	r = −0.01	r = −0.21	r = 0.02	r = 0.12	r = −0.19	r = −0.005	r = 0.06	r = −0.14
	*p* = 0.61	*p* = 0.12	*p* = 0.13	*p* = 0.27	*p* = 0.86	*p* = 0.11	*p* = 0.75	*p* = 0.24	*p* = 0.15	*p* = 0.95	*p* = 0.57	*p* = 0.29
Tanner 2–3	r = 0.11	r = 0.17	r = 0.10	r = 0.16	r = 0.19	r = 0.17	r = 0.15	r = 0.26	r = 0.15	r = 0.11	r = 0.18	r = 0.10
	*p* = 0.03	*p* = 0.02	*p* = 0.16	*p* < 0.01	*p* = 0.01	*p* = 0.01	*p* < 0.01	*p* < 0.001	*p* = 0.03	*p* = 0.02	*p* = 0.02	*p* = 0.14
Tanner 4–5	r = 0.07	r = −0.03	r = 0.20	r = 0.41	r = −0.35	r = 0.44	r = 0.21	r = 0.20	r = 0.21	r = 0.09	r = −0.04	r = 0.14
	*p* = 0.54	*p* = 0.83	*p* = 0.34	*p* < 0.001	*p* = 0.02	*p* = 0.03	*p* = 0.09	*p* = 0.21	*p* = 0.33	*p* = 0.47	*p* = 0.77	*p* = 0.52

BMI = Body mass index; ABSI = body shape index; TMI = triponderal mass index; ConI = conicity index VAI = visceral adiposity index; HOMA = homeostasis model assessment for insulin resistance; HOMA-β = HOMA of percentage β-cell function; QUICKI = quantitative insulin sensitivity check index; TyG-index = triglyceride and glucose index.

**Table 3:** Correlation between insulin resistance measurements and biochemical parameters.

Biochemical Parameters	Indirect Insulin Resistance Measurements											
	HOMA-IR			TyG-Index			HOMA-β			QUICK Index		
	All	Females	Males	All	Females	Males	All	Females	Males	All	Females	Males
Total cholesterol	r = 0.03	r = 0.03	r = 0.04	r = 0.28	r = 0.35	r = 0.23	r = 0.07	r = 0.07	r = 0.1	r = 0.01	r = 0.05	r = −0.006
	*p* = 0.37	*p* = 0.49	*p* = 0.42	*p* < 0.001	*p* < 0.001	*p* < 0.001	*p* = 0.06	*p* = 0.17	*p* = 0.06	*p* = 0.71	*p* = 0.37	*p* = 0.91
Tanner 1	r = −0.03	r = −0.03	r = −0.05	r = 0.22	r = 0.33	r = 0.06	r = 0.04	r = −0.003	r = 0.17	r = −0.05	r = −0.02	r = −0.11
	*p* = 0.63	*p* = 0.75	*p* = 0.68	*p* < 0.01	*p* < 0.01	*p* = 0.65	*p* = 0.57	*p* = 0.97	*p* = 0.19	*p* = 0.55	*p* = 0.84	*p* = 0.38
Tanner 2–3	r = 0.04	r = 0.16	r = −0.04	r = 0.30	r = 0.37	r = 0.26	r = 0.05	r = 0.14	r = 0.02	r = 0.02	r = 0.17	r = −0.05
	*p* = 0.43	*p* = 0.03	*p* = 0.53	*p* < 0.001	*p* < 0.001	*p* < 0.001	*p* = 0.29	*p* = 0.06	*p* = 0.72	*p* = 0.59	*p* = 0.02	*p* = 0.43
Tanner 4–5	r = 0.10	r = −0.20	r = 0.47	r = 0.32	r = 0.27	r = 0.26	r = 0.29	r = −0.08	r = 0.53	r = 0.07	r = −0.11	r = 0.34
	*p* = 0.43	*p* = 0.20	*p* = 0.02	*p* < 0.01	*p* = 0.08	*p* = 0.23	*p* = 0.02	*p* = 0.58	*p* = <0.01	*p* = 0.57	*p* = 0.49	*p* = 0.11
HDL-cholesterol	r = −0.16	r = −0.09	r = −0.25	r = −0.30	r = −0.26	r = −0.35	r = −0.21	r = −0.18	r = −0.28	r = −0.19	r = −0.12	r = −0.29
	*p* < 0.001	*p* = 0.08	*p* < 0.001	*p* < 0.001	*p* < 0.001	*p* < 0.001	*p* < 0.001	*p* = 0.001	*p* < 0.001	*p* < 0.001	*p* = 0.03	*p* < 0.001
Tanner 1	r = −0.13	r = −0.14	r = −0.11	r = −0.37	r = −0.36	r = −0.38	r = −0.07	r = −0.11	r = 0.01	r = −0.14	r = −0.14	r = −0.14
	*p* = 0.09	*p* = 0.18	*p* = 0.39	*p* < 0.001	*p* < 0.001	*p* < 0.01	*p* = 0.36	*p* = 0.31	*p* = 0.91	*p* = 0.08	*p* = 0.19	*p* = 0.27
Tanner 2–3	r = −0.19	r = −0.10	r = −0.33	r = −0.27	r = −0.23	r = −0.31	r = −0.25	r = −0.21	r = −0.33	r = −0.21	r = −0.10	r = −0.33
	*p* < 0.001	*p* = 0.18	*p* < 0.001	*p* < 0.001	*p* < 0.01	*p* < 0.001	*p* < 0.001	*p* < 0.01	*p* < 0.001	*p* < 0.001	*p* = 0.16	*p* < 0.001
Tanner 4–5	r = 0.06	r = −0.07	r = 0.09	r = −0.34	r = −0.30	r = −0.29	r = −0.11	r = −0.08	r = −0.12	r = −0.01	r = 0.005	r = −0.007
	*p* = 0.60	*p* = 0.65	*p* = 0.67	*p* < 0.01	*p* = 0.06	*p* = 0.18	*p* = 0.35	*p* = 0.59	*p* = 0.57	*p* = 0.89	*p* = 0.99	*p* = 0.97
Tryglicerides	r = 0.27	r = 0.23	r = 0.32	r = 0.88	r = 0.90	r = 0.87	r = 0.30	r = 0.31	r = 0.31	r = 0.30	r = 0.26	r = 0.33
	*p* < 0.001	*p* < 0.001	*p* < 0.001	*p* < 0.001	*p* < 0.001	*p* < 0.001	*p* < 0.001	*p* < 0.001	*p* < 0.001	*p* < 0.001	*p* < 0.001	*p* < 0.001
Tanner 1	r = 0.37	r = 0.31	r = −0.46	r = 0.93	r = 0.94	r = 0.94	r = 0.40	r = 0.42	r = 0.20	r = 0.38	r = 0.32	r = 0.46
	*p* < 0.001	*p* < 0.001	*p* < 0.001	*p* < 0.001	*p* < 0.001	*p* < 0.001	*p* < 0.001	*p* < 0.001	*p* = 0.12	*p* < 0.001	*p* < 0.01	*p* < 0.001
Tanner 2–3	r = 0.27	r = 0.27	r = −0.30	r = 0.88	r = 0.89	r = 0.88	r = 0.27	r = 0.27	r = 0.29	r = 0.28	r = 0.27	r = 0.29
	*p* < 0.001	*p* < 0.001	*p* < 0.001	*p* < 0.001	*p* < 0.001	*p* < 0.001	*p* < 0.001	*p* < 0.001	*p* < 0.001	*p* < 0.001	*p* < 0.001	*p* < 0.001
Tanner 4–5	r = 0.11	r = −0.09	r = 0.25	r = 0.94	r = 0.91	r = 0.95	r = 0.16	r = 0.19	r = 0.09	r = 0.13	r = 0.04	r = 0.20
	*p* = 0.38	*p* = 0.55	*p* = 0.25	*p* < 0.001	*p* < 0.001	*p* < 0.001	*p* = 0.19	*p* = 0.22	*p* = 0.68	*p* = 0.29	*p* = 0.80	*p* = 0.37
Systolic Blood Pressure	r = 0.34	r = 0.42	r = 0.27	r = 0.21	r = 0.22	r = 0.21	r = 0.25	r = 0.40	r = 0.12	r = 0.33	r = 0.47	r = 0.23
	*p* < 0.001	*p* < 0.001	*p* < 0.001	*p* < 0.001	*p* < 0.001	*p* < 0.001	*p* < 0.001	*p* < 0.001	*p* = 0.04	*p* < 0.001	*p* < 0.001	*p* < 0.001
Tanner 1	r = −0.37	r = 0.44	r = 0.24	r = 0.09	r = 0.19	r = −0.06	r = 0.28	r = 0.35	r = 0.13	r = 0.40	r = 0.47	r = 0.27
	*p* < 0.001	*p* < 0.001	*p* = 0.08	*p* = 0.27	*p* = 0.07	*p* = 0.66	*p* < 0.001	*p* < 0.01	*p* = 0.32	*p* < 0.001	*p* < 0.001	*p* = 0.04
Tanner 2–3	r = 0.25	r = 0.44	r = 0.13	r = 0.18	r = 0.22	r = 0.16	r = 0.17	r = 0.40	r = 0.02	r = 0.23	r = 0.44	r = 0.11
	*p* < 0.001	*p* < 0.001	*p* = 0.06	*p* < 0.001	*p* < 0.01	*p* = 0.02	*p* < 0.01	*p* < 0.001	*p* = 0.72	*p* < 0.001	*p* < 0.001	*p* = 0.13
Tanner 4–5	r = 0.38	r = 0.12	r = 0.68	r = 0.39	r = 0.27	r = 0.44	r = 0.11	r = 0.10	r = 0.09	r = 0.30	r = 0.19	r = 0.43
	*p* = 0.001	*p* = 0.44	*p* < 0.001	*p* < 0.01	*p* = 0.08	*p* = 0.03	*p* = 0.36	*p* = 0.50	*p* = 0.66	*p* = 0.01	*p* = 0.21	*p* = 0.04
Diastolic blood pressure	r = 0.22	r = 0.23	r = 0.23	r = 0.007	r = −0.06	r = 0.06	r = 0.19	r = 0.27	r = 0.13	r = 0.17	r = 0.21	r = 0.14
	*p* < 0.001	*p* < 0.001	*p* < 0.001	*p* = 0.85	*p* = 0.25	*p* = 0.26	*p* < 0.001	*p* < 0.001	*p* = 0.03	*p* < 0.001	*p* < 0.001	*p* = 0.02
Tanner 1	r = 0.27	r = 0.39	r = −0.05	r = −0.09	r = −0.03	r = −0.02	r = 0.17	r = 0.27	r = −0.11	r = 0.22	r = 0.33	r = −0.03
	*p* = 0.001	*p* < 0.001	*p* = 0.68	*p* = 0.27	*p* = 0.78	*p* = 0.08	*p* = 0.03	*p* = 0.01	*p* = 0.43	*p* < 0.001	*p* < 0.01	*p* = 0.78
Tanner 2–3	r = 0.14	r = 0.22	r = −0.08	r = −0.02	r = −0.09	r = −0.02	r = 0.14	r = 0.29	r = 0.03	r = 0.08	r = 0.16	r = −0.03
	*p* < 0.01	*p* < 0.01	*p* = 0.26	*p* = 0.71	*p* = 0.25	*p* = 0.78	*p* < 0.01	*p* < 0.001	*p* = 0.59	*p* = 0.11	*p* = 0.04	*p* = 0.63
Tanner 4–5	r = 0.28	r = −0.14	r = 0.73	r = 0.13	r = −0.004	r = 0.27	r = 0.08	r = −0.17	r = 0.33	r = 0.09	r = −0.22	r = 0.51
	*p* = 0.02	*p* = 0.37	*p* < 0.001	*p* = 0.29	*p* = 0.97	*p* = 0.21	*p* = 0.51	*p* = 0.28	*p* = 0.12	*p* = 0.45	*p* = 0.17	*p* = 0.01

BMI = Body mass index; ABSI = body shape index; TMI = triponderal mass index; ConI = conicity index VAI = visceral adiposity index; HOMA = homeostasis model assessment for insulin resistance; HOMA-β = HOMA of percentage β-cell function; QUICKI = quantitative insulin sensitivity check index; TyG-index = triglyceride and glucose index.

## Data Availability

Data reported in this study are available upon request from the corresponding author.
